# Value of image enhancement of endoscopic ultrasound for diagnosis of gastrointestinal subepithelial lesions

**DOI:** 10.1002/deo2.70026

**Published:** 2024-10-12

**Authors:** Hirofumi Yamazaki, Yasunobu Yamashita, Takaaki Tamura, Yuki Kawaji, Takashi Tamura, Masahiro Itonaga, Reiko Ashida, Toshio Shimokawa, Fumiyoshi Kojima, Keiji Hayata, Takao Maekita, Mikitaka Iguchi, Masayuki Kitano

**Affiliations:** ^1^ Second Department of Internal Medicine Wakayama Medical University Wakayama Japan; ^2^ Department of Human Pathology Wakayama Medical University Wakayama Japan; ^3^ Clinical Study Support Center Wakayama Medical University Wakayama Japan; ^4^ Second Department of Surgery Wakayama Medical University Wakayama Japan

**Keywords:** contrast‐enhanced harmonic EUS, EUS shear‐wave elastography, EUS strain elastography, GIST, subepithelial lesions

## Abstract

**Objectives:**

Among subepithelial lesions (SELs), gastrointestinal stromal tumors (GISTs) should be identified and surgically treated at an early stage. However, it is difficult to diagnose SELs smaller than 20 mm. In recent years, endoscopic ultrasound (EUS) elastography (EUS‐EG) and contrast‐enhanced harmonic EUS (CH‐EUS) have been reported to be useful for the diagnosis of SELs, although the diagnostic accuracy of a combination of EUS techniques with image enhancement is unknown.

**Methods:**

Patients with SELs who underwent EUS‐guided tissue acquisition, EUS shear‐wave elastography (EUS‐SWE), EUS strain elastography (EUS‐SE), and CH‐EUS from January 2019 to June 2023 were enrolled. To assess the diagnostic accuracy for differentiating GISTs from other SELs, shear‐wave velocity on EUS‐SWE, the strain ratio on EUS‐SE, and vascularity on CH‐EUS were determined and their diagnostic accuracies were compared.

**Results:**

Forty‐three patients were enrolled. When the cut‐off value was set at 3.27 m/s, the sensitivity, specificity, and diagnostic accuracy of shear‐wave velocity were 28.6%, 86.2%, and 34.9%, respectively. When the cut‐off value was set at 3.79, the sensitivity, specificity, and diagnostic accuracy of the strain ratio were 93.1%, 64.3%, and 83.7%, respectively. The sensitivity, specificity, and diagnostic accuracy of CH‐EUS were 79.3%, 92.3%, and 83.7%, respectively. When EUS‐SE was combined with CH‐EUS, the sensitivity and diagnostic accuracy were the highest among binary combinations of image enhancement modalities.

**Conclusions:**

EUS‐SE and CH‐EUS are useful for differentiating GISTs from other SELs. Furthermore, the use of both modalities may further improve the identification of GISTs.

## INTRODUCTION

The characterization of subepithelial lesions (SELs) is important for determining their optimal treatment strategy. It is essential to differentiate gastrointestinal stromal tumors (GISTs) from other SELs because GISTs have malignant potential. Although the annual incidence of GISTs is approximately 10 patients per million people,^1^ early diagnosis of GISTs is important. Surgical resection is recommended when a GIST is diagnosed.^2^ Endoscopic ultrasound (EUS)‐guided tissue acquisition (EUS‐TA) is performed to diagnose SELs, with a reported diagnostic rate of 84%,^3^ but the diagnostic rate for SELs smaller than 20 mm is lower at 53.9%–62.0%.^4^, ^5^

EUS elastography (EUS‐EG) has been performed as a minimally invasive examination in recent years. EUS‐EG includes EUS shear‐wave elastography (EUS‐SWE) and EUS strain elastography (EUS‐SE). There are two reports on the role of EUS‐SE in the diagnosis of SELs.^6^, ^7^ Tsuji et al. reported the usefulness of EUS‐SE for differentiating GIST from other SELs by using elasticity scores. Although GIST tended to be harder than other SELs in the previous reports, they did not evaluate the diagnostic ability of EUS‐SE for diagnosis of GIST. While there have been reports of EUS‐SE, there have been no reports of the usefulness of EUS‐SWE for the diagnosis of GISTs. Furthermore, no report has compared tissue hardness measured by EUS‐EG with the degree of fibrosis in resected specimens.

In a previous study, GISTs showed 100% hyperenhancement at the early phase and 87.5% of non‐GISTs showed non‐hyperenhancement on contrast‐enhanced harmonic EUS (CH‐EUS).^8^ A recent meta‐analysis reported the value of CH‐EUS in gastrointestinal diseases, particularly for distinguishing GISTs from benign SELs.^9^ It reported that pooled sensitivity was 89% (95% confidence interval [CI] 0.82–0.93), specificity was 82% (95% CI 0.66–0.92), and the area under the receiver operating characteristic curve (AUROC) was 0.89 to discriminate between GISTs and benign SELs. These results suggested that CH‐EUS is useful for characterizing SELs. However, no report has compared its diagnostic ability with that of EUS‐EG.

The purpose of this retrospective study was to compare the usefulness of EUS‐SWE, EUS‐SE, and CH‐EUS for the differentiation of GISTs. The present study also aimed to evaluate the diagnostic ability of a combination of EUS‐EG and CH‐EUS for the differentiation of GISTs. Furthermore, the present study aimed to investigate the correlation between tissue hardness measured by EUS‐EG and the degree of fibrosis in pathological tissue specimens.

## METHODS

### Patients

Patients with SELs who underwent EUS‐TA, EUS‐SWE, EUS‐SE, and CH‐EUS from January 2019 to June 2023 were enrolled. Written informed consent was obtained from individuals.

### Study endpoints

The primary endpoint was to evaluate and compare the diagnostic abilities of EUS‐SWE, EUS‐SE, and CH‐EUS for all patients with SELs. The secondary endpoint was to correlate the degree of fibrosis in surgical specimens of GISTs with tissue hardness determined by EUS‐EG.

### Endoscopic procedure

Convex‐type endoscopes (GF‐UCT260; Olympus) with ultrasound observation systems (ARIETTA 850; Hitachi Aloka Medical, Ltd.) were utilized for EUS‐SWE, EUS‐SE, and CH‐EUS. A conventional EUS examination was initially performed for patients sedated with midazolam. The EUS scope was inserted after sedation. EUS‐SWE, EUS‐SE, and CH‐EUS were performed before EUS‐TA when conventional EUS depicted SELs.

### EUS‐shear‐wave elastography

**FIGURE 1:**
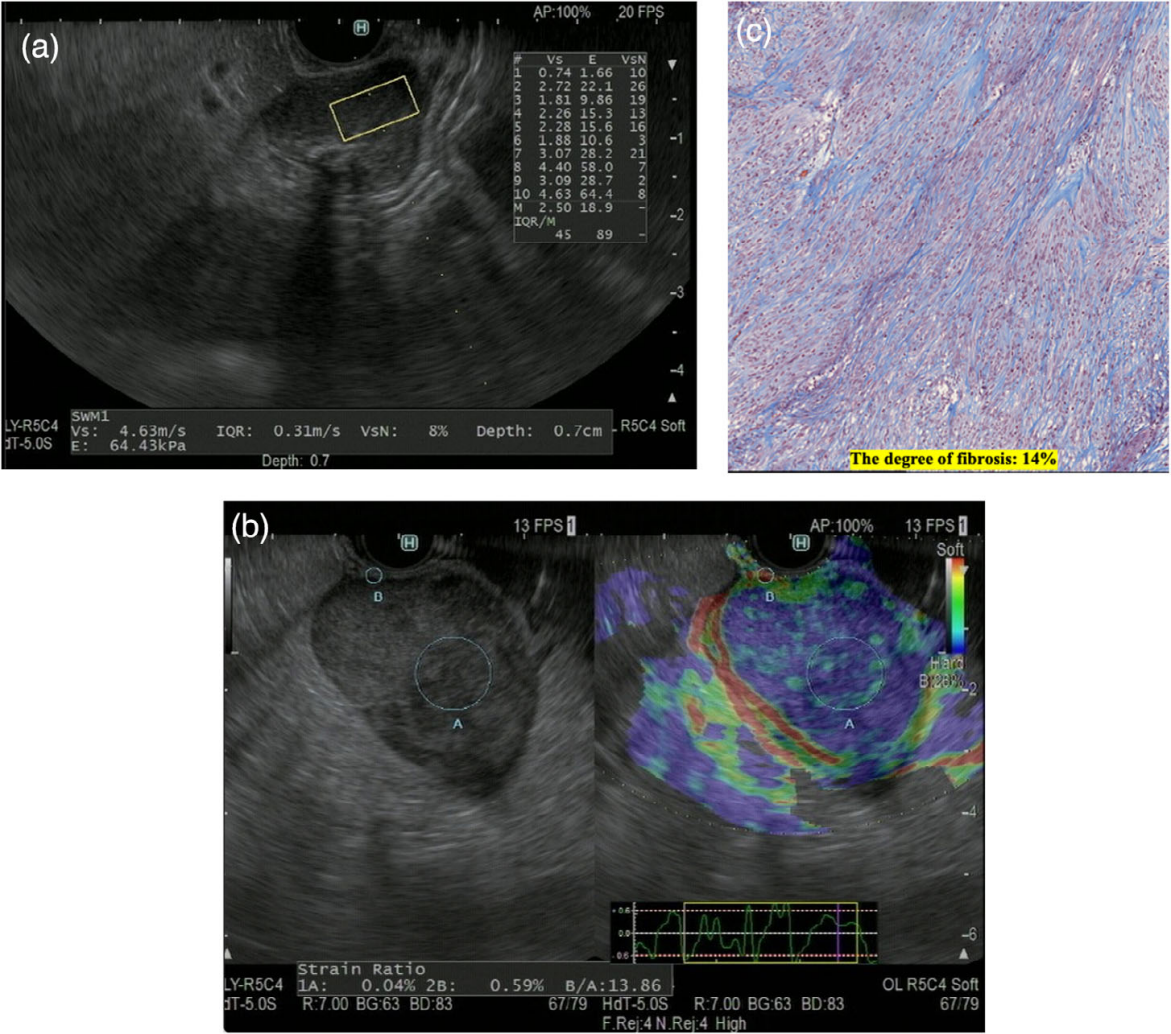
(a) Image of a subepithelial lesion (SEL) examined by endoscopic ultrasound shear‐wave elastography (EUS‐SWE). Shear‐wave velocity (Vs) was measured 10 times in the present study. (b) Image of a SEL examined by endoscopic ultrasound strain elastography (EUS‐SE). The strain ratio was measured twice in the present study. Evaluation of images near the apex of the graph waveforms is desirable, and as far as possible images of the apex of the graph were considered. (c) Image of a pathology specimen stained with Masson's trichrome (×400). A BZ‐800 microscope (Keyence Corporation) was utilized to measure the degree of fibrosis in resected specimens.

The region of interest (ROI) was set at the maximum diameter of SELs, avoiding vessels. The size of the ROI was 5×10 mm. Shear‐wave velocity (Vs) and the percentage of the net amount of effective Vs (VsN) were measured by EUS‐SWE. The VsN value showed which percentage of the measurement value was used in the calculation of Vs. The VsN value was used to assess whether or not the Vs value was reliable. Vs values were measured 10 times and the mean Vs value was determined for each SEL (Figure 1a). If the measurement was not satisfactory, Vs values were measured again and only 10 reliable Vs values were used for evaluation. Each EUS‐SWE result was evaluated by examining each EUS‐SWE image retrospectively.

### EUS‐strain elastography

For the ROI setting, we referred to a previous report by Garcia et al.^10^ Two different areas were selected for quantitative elastographic analysis. Area A was selected so that it included as much of the target SEL as possible without including the surrounding tissues. Area B was selected within the gastrointestinal wall excluding the target SEL. The strain ratio of the elastographic evaluation was defined as the quotient B/A (Figure 1b). Strain indicators indicate the degree of pressure intensity and strain, and when the graph shows maximum amplitude under pressure from aortic pulsation, it is judged to be an image drawn with the desired vibration energy. Evaluation of images near the apex of the graph waveforms is desirable, and as far as possible images of the apex of the graph were considered. Each EUS‐SE result was evaluated using each EUS‐SE still image in which the maximum diameter of the SEL was depicted. The strain ratio was measured twice for each SEL.

### Contrast‐enhanced harmonic‐EUS

Sonazoid (GE Healthcare) was used as an ultrasound contrast agent for CH‐EUS. After the sonazoid is injected into a peripheral vein, ultrasound waves disrupt them and produce a signal. SELs were evaluated to determine whether they displayed hyperenhancement or non‐hyperenhancement. In this study, we defined SELs of hypervascular enhancement at the early phase as GISTs. After recording the CH‐EUS videos, vascular patterns were independently assessed by three endosonographers with at least 10 years of EUS experience. These assessments were made without knowledge of the final diagnosis. When the three endosonographers arrived at different conclusions, the consensus verdict of the two endosonographers was adopted. Typical examples of hypervascular enhancement (GIST case) and non‐hypervascular enhancement (leiomyoma case) are shown in Videos [Link], [Link], respectively.

### Combinations of image enhancement modalities

We used binary combinations of EUS‐SE, EUS‐SWE, and CH‐EUS to distinguish GISTs from other SELs. Three combinations (EUS‐SE/EUS‐SWE, EUS‐SE/CH‐EUS, and EUS‐SWE/CH‐EUS) were assessed. If either of the two modalities in a combination was positive for GIST, then the combination was considered positive for GIST.

### Histological evaluation

The final diagnosis of SELs was based on EUS‐TA or pathological evaluation of resected specimens. The histological diagnosis of GISTs was defined based on the presence of SELs composed of spindle cells that were positively stained for c‐kit, CD34, and/or DOG‐1. In cases where EUS‐TA revealed non‐GISTs or did not provide a definitive diagnosis, patients who were followed up for 1 year and showed no significant change in SELs were defined as having non‐GISTs. The areas of histological fibrosis were measured separately using imaging software (BZ‐X800; Keyence Corporation) and Masson's trichrome staining of specimens (Figure 1c). The degree of fibrosis was measured at the location with the most fibrosis in a 400× field of view imaged with a BZ‐800 microscope.

### Statistical analysis

Fisher's exact test for qualitative variables and the Mann‐Whitney U test for quantitative variables were used to compare categorical variables. The diagnostic accuracies of Vs and the strain ratio for SELs were calculated from the ROC curves, with the maximum Youden index used to determine cut‐off points, and sensitivity and specificity were calculated. AUROC values were defined as low (i.e., 0.5–<0.7), moderate (i.e., 0.7–<0.9), or high (i.e., ≥0.9) accuracy. Correlations between the elasticity values and the degree of fibrosis were evaluated using Spearman's correlation. All statistical analyses were performed using JMP Pro version 13 (SAS Institute Inc.). A *p*‐value <0.05 was considered statistically significant. Means and standard deviations are provided throughout this report.

## RESULTS

### Patient characteristics

Forty‐three patients were enrolled in this study. Patient details are provided in Table 1. Final diagnoses included GIST, leiomyoma, aberrant pancreas, lipoma, and schwannoma in 29, eight, four, one, and one patients, respectively. Surgical resection was performed in 27 patients with GISTs and one patient with leiomyoma.

**TABLE 1:** Patient characteristics in the gastrointestinal stromal tumor and non‐gastrointestinal stromal tumor groups.^†^

Variable	GIST (*n* = 29)	Non‐GIST (*n* = 14)	*p*‐value
Age, years (mean ± SD)	67.8 ± 12.9	61.2 ± 15.2	0.15
Sex (*n*), male/female	17/12	7/7	0.75
Size, mm (mean ± SD)	27.6 ± 15.2	21.3 ± 9.3	0.16
Lesion site (*n*)			
Gastric /non‐gastric	29/0	12/2	0.10
Gastric site			
Cardia	2	3	
Fornix	5	1	
Body	21	7	
Antrum	1	1	
Non‐gastric site			
Esophagus	0	1	
Duodenum	0	1	
Modified Fletcher classification			
Very low risk	9	‐	
Low risk	13	‐	
Intermediate risk	3	‐	
High risk	2	‐	
Unknown (no surgical resection)	2	‐	

^†^ All GIST cases were positive for c‐kit. All leiomyoma cases were negative for c‐kit and positive for desmin, and the schwannoma case was c‐kit negative and S‐100 positive.

Abbreviation: GIST, gastrointestinal stromal tumor.

### EUS‐shear‐wave elastography

**FIGURE 2:**
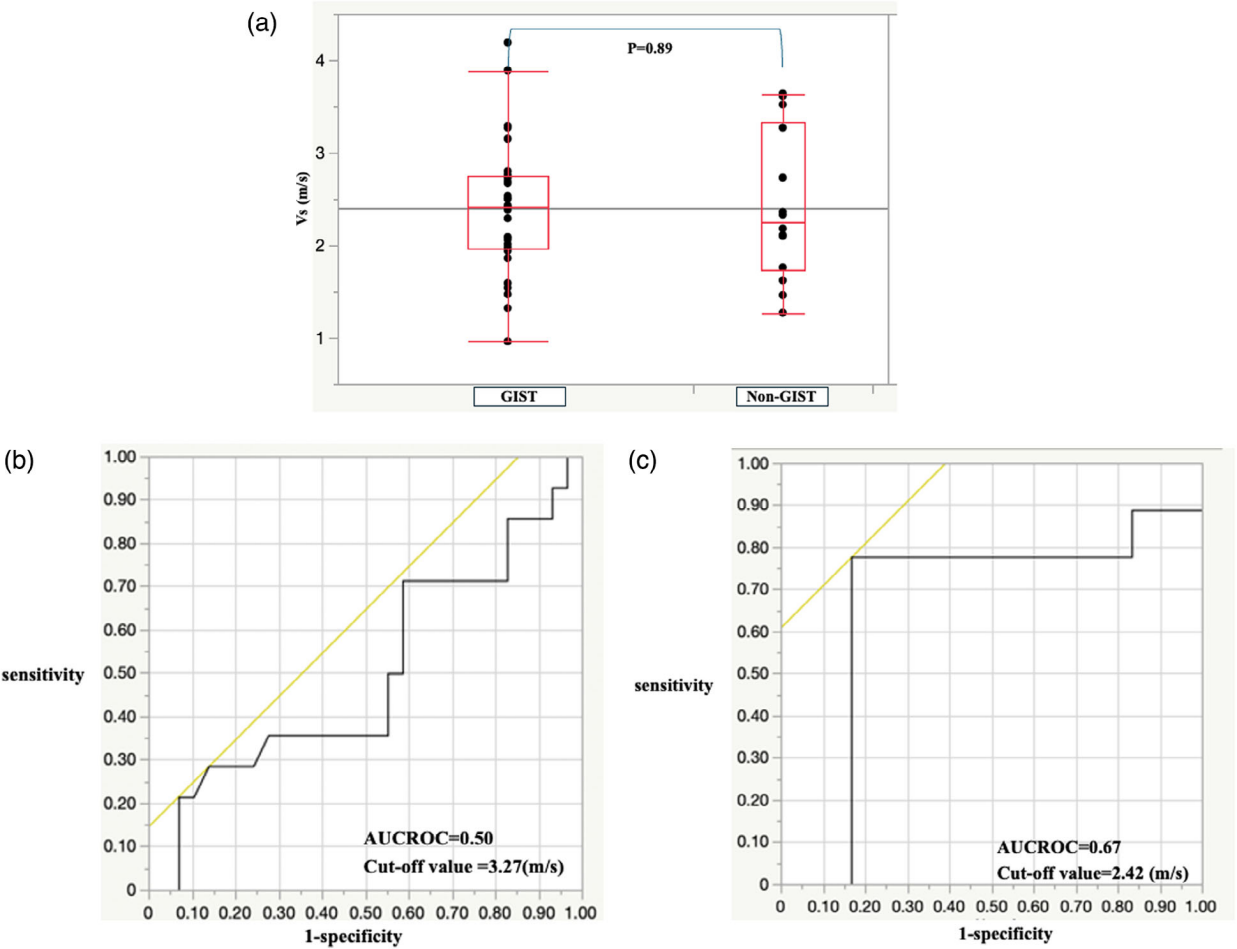
Shear‐wave velocity (Vs) results: (a) Box‐and‐whisker plot of Vs in the gastrointestinal stromal tumor (GIST) and non‐GIST groups. (b) Receiver operating characteristic (ROC) curve for the differential diagnosis of GISTs and non‐GISTs using Vs. (c) ROC curve for the differential diagnosis of GISTs and non‐GISTs smaller than 20 mm using Vs.

For all SELs, Vs was 2.39 ± 0.73 and 2.42 ± 0.81 m/s in the GIST and non‐GIST groups, respectively (Table 2). The mean Vs values for leiomyomas and aberrant pancreas were 2.92 and 1.71 m/s, respectively. The Vs values for lipoma and schwannoma were 1.62 and 2.10 m/s, respectively. Vs value did not significantly differ between the two groups (*p* = 0.89, Figure 2a). When the cut‐off value was set at 3.27 m/s, the sensitivity, specificity, diagnostic accuracy, and AUROC of Vs were 28.6%, 86.2%, 34.9%, and 0.50, respectively (Table 3 and Figure 2b). The mean VsN value was 28.7 ± 22.8% and 44.0 ± 24.9% in the GIST and non‐GIST groups, respectively (Table 2). For SELs smaller than 20 mm, the sensitivity, specificity, diagnostic accuracy, and AUROC of Vs were 77.8%, 83.3%, 80.0%, and 0.67, respectively, when the cut‐off value was set at 2.42 m/s (Table 3 and Figure 2c). The diagnostic accuracy was 34.9% for all SELs, although the value was 80.0% for SELs smaller than 20 mm. The mean VsN values were 25.7 ± 12.3% and 47.7 ± 16.5% in the GIST and non‐GIST groups, respectively, for SELs smaller than 20 mm. (Table 2).

**TABLE 2:** Values of gastrointestinal stromal tumors and non‐gastrointestinal stromal tumors measured by endoscopic ultrasound elastography.

	GIST (*n* = 29)	Non‐GIST (*n* = 14)	*p*‐value
**All SELs**			
Vs (m/s), mean ± SD	2.39 ± 0.73	2.42 ± 0.81	0.89
VsN (%), mean±SD	28.7 ± 22.8	44.0 ± 24.9	0.06
Strain ratio, mean±SD	8.15 ± 5.22	4.56 ± 3.97	0.03

	GIST (*n* = 9)	Non‐GIST (*n* = 6)	*p‐*value
**<20 mm SELs**			
Vs (m/s), mean ± SD	2.38 ± 0.72	2.23 ± 0.71	0.69
VsN (%), mean ± SD	25.7 ± 12.3	47.7 ± 16.5	0.01
Strain ratio, mean ± SD	8.13 ± 5.03	3.62 ± 2.07	0.06

Abbreviations: EUS‐EG, endoscopic ultrasound elastography; GIST, gastrointestinal stromal tumor; SEL, subepithelial lesion; Vs, shear‐wave velocity; VsN, net amount of effective shear‐wave velocity.

**TABLE 3:** Sensitivity, specificity, and diagnostic accuracy of each modality and their combinations for diagnosis of gastrointestinal stromal tumors.

	Cut‐off value	Sensitivity (%)	Specificity (%)	Diagnostic accuracy (%)	AUROC
**All SELs**					
EUS‐SWE (Vs value) alone	3.27 m/s	28.6	86.2	34.9	0.50
EUS‐SE (strain ratio) alone	3.79	93.1	64.3	83.7	0.78
CH‐EUS alone	‐	79.3	92.3	83.7	‐
EUS‐SWE+EUS‐SE	‐	93.1	50.0	79.1	‐
EUS‐SWE+CH‐EUS	‐	86.2	64.3	79.1	‐
EUS‐SE+CH‐EUS	‐	96.7	64.3	86.0	‐
**<20 mm SELs**					
EUS‐SWE (Vs value) alone	2.42 m/s	77.8	83.3	80.0	0.67
EUS‐SE (strain ratio) alone	4.42	88.9	83.3	86.7	0.81
CH‐EUS alone	‐	88.9	83.3	93.3	‐
EUS‐SWE+EUS‐SE	‐	100	83.3	93.3	‐
EUS‐SWE+CH‐EUS	‐	100	66.7	86.7	‐
EUS‐SE+CH‐EUS	‐	100	66.7	86.7	‐

Abbreviations: AUROC, area under the receiver operating characteristic curve; CH‐EUS, contrast‐enhanced harmonic endoscopic ultrasound; EUS‐SE, endoscopic ultrasound strain elastography; EUS‐SWE, endoscopic ultrasound shear‐wave elastography; GIST, gastrointestinal stromal tumor; Vs, shear‐wave velocity.

### EUS‐strain elastography

**FIGURE 3:**
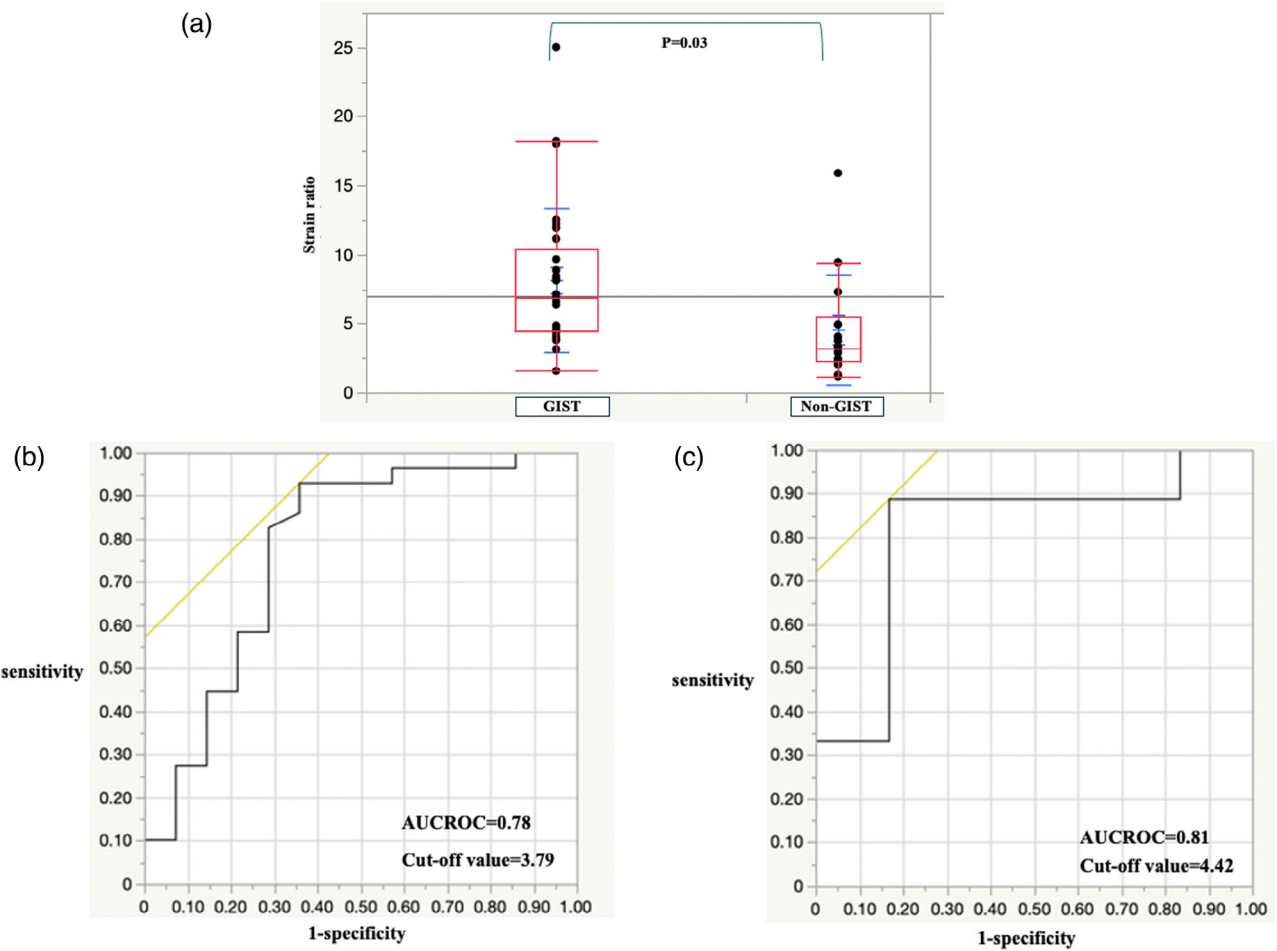
Strain ratio results: (a) Box‐and‐whisker plot of the strain ratio in the gastrointestinal stromal tumor (GIST) and non‐GIST groups. (b) Receiver operating characteristic (ROC) curve for the differential diagnosis of GISTs and non‐GISTs using the strain ratio. (c) ROC curve for the differential diagnosis of GISTs and non‐GISTs smaller than 20 mm using the strain ratio.

For all SELs, the strain ratio was 8.15 ± 5.22 and 4.56 ± 3.97 in the GIST and non‐GIST groups, respectively (Table 2). The mean strain ratios for leiomyoma and aberrant pancreas were 4.18 and 5.56, respectively. The strain ratios for lipoma and schwannoma were 3.31 and 4.92, respectively. The strain ratio significantly differed between the two groups (*p* = 0.03, Figure 3a). When the cut‐off value was set at 3.79, the sensitivity, specificity, diagnostic accuracy, and AUROC of the strain ratio were 93.1%, 64.3%, 83.7%, and 0.78, respectively (Table 3 and Figure 3b). For SELs smaller than 20 mm, the sensitivity, specificity, diagnostic accuracy, and AUROC of the strain ratio were 88.9%, 83.3%, 86.7%, and 0.81, respectively, when the cut‐off value was set at 4.42 (Table 3 and Figure 3c).

### Contrast‐enhanced harmonic‐EUS

On CH‐EUS, 23 of 29 GIST cases showed hyperenhancement and 13 of 14 non‐GIST cases showed non‐hyperenhancement (Table 4). For all SELs, the sensitivity, specificity, and diagnostic accuracy of CH‐EUS were 79.3%, 92.3%, and 83.7%, respectively (Table 3). For SELs smaller than 20 mm, eight of nine GIST cases showed hyperenhancement and five of six non‐GIST cases showed non‐hyperenhancement (Table 4). For SELs smaller than 20 mm, the sensitivity, specificity, and diagnostic accuracy of CH‐EUS were 88.9%, 83.3%, and 93.3%, respectively (Table 3).

**TABLE 4:** Enhancement of gastrointestinal stromal tumors and non‐gastrointestinal stromal tumors on contrast‐enhanced harmonic endoscopic ultrasound.

	GIST (*n* = 29)	Non‐GIST (*n* = 14)
**(Overall patients)**		
Hyperenhancement (%)	23/29 (79.3)	1/14 (7.1)
Non‐hyperenhancement (%)	6/29 (20.7)	13/14 (92.9)

	GIST (*n* = 9)	Non‐GIST (*n* = 6)
**(<20 mm patients)**		
Hyperenhancement (%)	8 (88.9)	1 (16.7)
Non‐hyperenhancement (%)	1 (11.1)	5 (83.3)

Abbreviations: CH‐EUS, contrast‐enhanced harmonic endoscopic ultrasound; GIST, gastrointestinal stromal tumor.

### Combinations of image enhancement modalities

For all SELs, the sensitivity, specificity, and diagnostic accuracy of a combination of EUS‐SWE and EUS‐SE were 93.1%, 50.0%, and 79.1%, respectively (Table 3). For all SELs, the sensitivity, specificity, and diagnostic accuracy of a combination of EUS‐SWE and CH‐EUS were 86.2%, 64.3%, and 79.1%, respectively (Table 3). For all SELs, the sensitivity, specificity, and diagnostic accuracy of a combination of EUS‐SE and CH‐EUS were 96.7%, 64.3%, and 86.0%, respectively (Table 3).

For SELs smaller than 20 mm, the sensitivity, specificity, and diagnostic accuracy of a combination of EUS‐SWE and EUS‐SE were 100%, 83.3%, and 93.3%, respectively (Table 3). For SELs smaller than 20 mm, the sensitivity, specificity, and diagnostic accuracy of a combination of EUS‐SWE and CH‐EUS were 100%, 66.7%, and 86.7%, respectively (Table 3). For SELs smaller than 20 mm, the sensitivity, specificity, and diagnostic accuracy of a combination of EUS‐SE and CH‐EUS were 100%, 66.7%, and 86.7%, respectively (Table 3).

### Correlation of tumor elasticity and pathological findings

**FIGURE 4:**
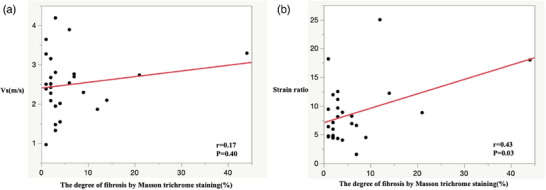
Correlations between the degree of fibrosis and tissue hardness values of gastrointestinal stromal tumors (GISTs). (a) Correlation between the degree of fibrosis and shear‐wave velocity (Vs) for overall GIST cases. (b) Correlation between the degree of fibrosis and the strain ratio for overall GIST cases.

In GIST cases that underwent surgical resection, no clear correlation was found between Vs and the degree of fibrosis (*r* = 0.17, *p* = 0.40; Figure 4a). On the other hand, a weak correlation was observed between the strain ratio and the degree of fibrosis (*r* = 0.43 *p* = 0.03; Figure 4b).

### Relationship between the degree of fibrosis in GIST and the contrast effect

On CH‐EUS, 23 surgically resected GISTs were hypervascular and four were non‐hypervascular. In the resected GIST cases, the mean degrees of fibrosis of the hypervascular enhancement group and non‐hypervascular enhancement group were 4.61 ± 4.97% and 15.0 ± 19.5%, respectively. The degree of fibrosis tended to be higher in the non‐hypervascular enhancement group.

## DISCUSSION

This is the first study to compare the usefulness of EUS‐SWE and EUS‐SE for the diagnosis of SELs. In AUROC analysis, EUS‐SE had a better diagnostic performance for SELs than EUS‐SWE, as well as higher sensitivity. Previous studies with EUS‐SE reported that GISTs are harder than non‐GISTs.^11^, ^12^ The results of the present study are consistent with those of these previous studies. Vs values for SELs smaller than 20 mm could not be accurately measured because the ROI for EUS‐SWE (5 × 10 mm) was larger than the whole sizes of some small SELs. This might have resulted in an overestimation of the diagnostic accuracy for SELs smaller than 20 mm.

EUS‐SE and EUS‐SWE allow quantitative analysis of tumor tissue hardness, which is thought to be more objective than analysis of tumor structure on EUS imaging. However, it was unclear whether these hardness values reflect tumor hardness. To determine whether EUS‐SWE or EUS‐SE adequately reflects the pathological findings, correlations between the degree of fibrosis and tissue hardness values were investigated using resected GIST specimens. To the best of our knowledge, there is no report of correlations between tissue hardness measured by EUS‐EG and the degree of fibrosis in histopathology specimens of GISTs. The strain ratio moderately correlated with the degree of fibrosis in pathology specimens and was considered to adequately reflect fibrosis in GISTs. On the other hand, tissue hardness measured by EUS‐SWE did not clearly correlate with the degree of fibrosis. These results suggest that EUS‐SE can be used to measure adequately tissue hardness, whereas EUS‐SWE cannot. The reason for this is thought to be related to the VsN value. Ohno et al.^13^ reported that VsN values are lower in pancreatic cancer than in non‐pancreatic cancer lesions. This may be due to fibrosis and necrosis inside pancreatic cancer tumors, which may result in poor Vs measurement. In this study, VsN was lower in the GIST group than the non‐GIST group for all SELs (Table 2). For SELs smaller than 20 mm, VsN was also lower in the GIST group. This suggests that Vs was not well measured in GISTs. Furthermore, differences in the ROI were also thought to affect the results because the size of the ROI could not be changed in EUS‐SWE, but could be adjusted to encompass the entire tumor in EUS‐SE.

In CH‐EUS, the diagnosis of SELs is based on the difference in contrast effect. In the present study, hyperenhancement was more common in the GIST group and non‐hyperenhancement was more common in the non‐GIST group as previously reported.^14^ In this study, the degree of fibrosis tended to be higher in the non‐hypervascular enhancement group. If the contrast effect is non‐hypervascular enhancement with CH‐EUS, it may be a GIST with strong fibrosis, and EUS‐SE may be able to diagnose GISTs that are difficult to diagnose with CH‐EUS. However, to confirm this, it will be necessary to increase the number of cases because the number of non‐hypervascular enhancement cases in the present study was small.

In this study, combinations of EUS‐SWE and EUS‐SE, EUS‐SWE and CH‐EUS, and EUS‐SE and CH‐EUS were performed. The combination of EUS‐SE and CH‐EUS showed improved diagnostic accuracy.

EUS‐TA is a diagnostic method with high diagnostic performance for SELs. However, the diagnostic accuracy with EUS‐TA for SELS smaller than 20 mm was low.^4^, ^5^ Compared with EUS‐TA, EUS‐SE, and CH‐EUS may be relatively minimally invasive methods for diagnosis of SELs. Performing CH‐EUS or EUS‐SE for all SELs and SELs smaller than 20 mm resulted in high diagnostic accuracy, and the combination of CH‐EUS and EUS‐SE also resulted in high diagnostic accuracy. Therefore, these two modalities can be used to complement EUS‐TA for the differentiation of GISTs from other types of SELs.

This study has several limitations. First, it was a retrospective observational study. Second, detailed pathological evaluation could not be performed because the number of cases was small and 15 of the 43 patients did not undergo surgical resection. Finally, the final diagnoses were based on immunostaining results of all SELs except lipoma and aberrant pancreas, and we did not analyze for gene mutations in EUS‐TA samples. Therefore, GISTs might have been included in some SELs that were negative for c‐kit.

In conclusion, tissue hardness measured by EUS‐SE modestly correlated with tumor fibrosis and was considered to adequately represent tissue hardness in GISTs. EUS‐SE reflected the pathology better than EUS‐SWE and had superior diagnostic accuracy for GISTs. The combination of EUS‐SE and CH‐EUS was more diagnostically accurate than EUS‐SE alone or CH‐EUS alone. On the other hand, for the diagnosis of SELs smaller than 20 mm, the accuracies of the three modalities and their binary combinations were higher than 80%, indicating that they could have the potential to complement EUS‐TA.

## CONFLICT OF INTEREST STATEMENT

Masayuki Kitano has received honoraria from Olympus Corporation for delivering lectures at conferences and has received research grants from Boston Scientific Corporation and Medico's Hirata Incorporated. The other authors declare no conflict of interest.

## ETHICS STATEMENT

The present study was approved by the ethics committee of Wakayama Medical University (No. 4070) and was performed in accordance with the ethical standards laid down in the 1964 Declaration of Helsinki and its later amendments.

## PATIENT INFORMED CONSENT

Written informed consent was obtained from individuals.

## Supporting information

Video S1 A typical GIST case with hypervascular enhancement on CH‐EUS.

Video S2 A typical leiomyoma case with non‐hypervascular enhancement on CH‐EUS.
